# Religious faith, gratitude, conflict resolution styles, and romantic love

**DOI:** 10.3389/fsoc.2025.1588365

**Published:** 2025-06-27

**Authors:** Marius Marici, Adelia Furdui (Florea), Patricia Runcan

**Affiliations:** ^1^Faculty of Psychology and Education Sciences, Ştefan cel Mare University, Suceava, Romania; ^2^Department of Social Work, Faculty of Sociology and Psychology, West University of Timişoara, Timişoara, Romania

**Keywords:** religious faith, gratitude, conflict resolution, romantic love, AMOS

## Abstract

**Background:**

The study emphasizes the value of integrating psychological and spiritual dimensions in understanding relational harmony. The aim of this research was to investigate the role of gratitude and conflict resolution styles as mediators between religious faith and romantic love.

**Methods:**

A structural equation model was performed with data from married males and females from Romania (*N* = 226, M_age_ = 40.67, SD_age_ = 11.76). Established measurement tools assessed key variables, while the model's validity was evaluated through multiple statistical benchmarks (e.g., fit indices), allowing a comprehensive assessment of pathways linking religious faith, gratitude, conflict resolution, and romantic love.

**Results:**

Analyses indicated that religious faith strongly predicted gratitude. Gratitude, in turn, had a favorable impact on cooperative conflict-resolution behaviors, which were closely linked to strengthened romantic love. The model exhibited strong validity.

**Conclusions:**

The findings underscore the central role of intrinsic spiritual values and gratitude in fostering effective conflict management and enriching romantic love. These insights highlight potential applications in therapeutic settings and relationship-building programs, suggesting that fostering these personal qualities could enhance partnership satisfaction.

## 1 Introduction

In a world where inner convictions shape our daily lives, religious faith represents a vital foundation that not only nurtures a sense of purpose but also cultivates personal gratitude. Drawing on the rich tapestry of religious practice—from prayer and worship to the internalization of spiritual values—research has frequently demonstrated that individuals with a deep, intrinsic commitment to their faith experience heightened gratitude and manifest positive outcomes (Emmons and Kneezel, 2005; Krause, 2009; Bahnaru et al., 2019).

From a theoretical perspective, Attachment theory (Bowlby, 1969) integrated with Relational Capital Theory (Nahapiet and Ghoshal, 1998) explains the association between religious faith and interpersonal relationships. Religious faith provides individuals with “spiritual capital” (psychological, emotional, and moral resources), helping them more frequently experience positive emotions such as gratitude. This, in turn, facilitates the adoption of constructive conflict resolution styles. These constructive styles improve stability and satisfaction in romantic love by strengthening attachment and enhancing the quality of interpersonal interactions. In essence, the process is: Religious Faith → enhances → Gratitude → promotes → Constructive Conflict Resolution Styles → supports → Stable and Satisfying Romantic Love (Mikulincer and Shaver, 2010; Kale et al., 2000). This perspective has been relatively understudied empirically, although studies generally have demonstrated that personality traits and individual choices influence interpersonal relationships. The present research aimed at investigating how religious faith influences sequentially gratitude, conflict resolution, and romantic love.

### 1.1 Religious faith and gratitude

Research has often shown that religious faith is associated with higher levels of personal gratitude. Generally, religious people are more grateful (Sandage et al., 2011), although there were less studies on the effect of faith on gratitude as more often studies investigated the effect of gratitude on wellbeing (Kraus et al., 2015). A study found that individuals with a strong internalized religious orientation—who are more likely to view their life's blessings as gifts from a benevolent God—tend to report higher levels of gratitude (Emmons and Kneezel, 2005). Similarly, religious practices such as prayer and participation in worship are linked with enhanced feelings of thankfulness (Krause, 2009).

However, the strength of this relationship seems not to be uniform across all studies or populations. Some investigations have reported non-significant associations under certain conditions. For instance, in samples where religious involvement is primarily extrinsic (i.e., engaged for social or utilitarian reasons rather than internal conviction), the correlation between religious faith and personal gratitude is often weaker or non-significant (Huynh et al., 2024) Moreover, cross-cultural research has sometimes failed to replicate a strong positive link; for example, when comparing religious and non-religious individuals across different cultural contexts, some researchers have found that the predicted relationship does not reach significance, suggesting that the influence of religious faith on gratitude may be moderated by cultural factors or the way religious practice is conceptualized (Watkins et al., 2024). As a result, the following hypothesis was established: H1: “Religious faith will positively influence personal gratitude.”

### 1.2 Gratitude and conflict resolution styles in couple

Research indicates that gratitude interventions can foster more adaptive conflict-resolution strategies in interpersonal relationships. For example, one study on gratitude journaling in intimate dyadic relationships found that participants who engaged in daily gratitude writing reported an increased use of positive problem-solving techniques and a reduced reliance on withdrawal or aggressive responses during conflicts (Dizon, 2020).

In a study Algoe et al. (2010) investigated how everyday expressions of gratitude between partners can boost relationship quality. Although their primary focus was on romantic relationships, their findings suggest that the positive emotional experience associated with gratitude can help individuals reframe interpersonal challenges and engage in more constructive problem-solving during conflicts. Their research indicates that when people notice and express gratitude, they tend to experience more positive moods and interpersonal warmth, which can serve as a buffer against negative interactions and promote more adaptive conflict-resolution styles.

Another article (Algoe et al., 2010) indicates that everyday expressions of gratitude help couples feel more understood and valued. In qualitative interviews, participants described how noticing and expressing gratitude shifted their focus away from conflicts and toward cooperative problem solving. They reported that gratitude increased their empathy for one another and opened the lines of communication, thereby making it easier to adopt a “win–win” approach during disagreements.

Systematic reviews of gratitude interventions in workplace settings have revealed mixed results—while such interventions consistently reduce stress and depressive symptoms, their impact on specific conflict management behaviors appears inconsistent (Komase et al., 2021).

Moreover, contextual factors such as the type of relationship and cultural norms seem to moderate these effects; gratitude's positive influence on conflict resolution is generally more pronounced in intimate, emotionally connected relationships than in more formal or organizational environments. Fehr et al. (2017) developed a multilevel model of gratitude in the workplace and argued that the positive influence of gratitude on conflict resolution is generally attenuated in such formal settings, where the relational bonds are less intense. Together, these studies indicate that contextual factors such as the nature of the relationship and prevailing cultural norms moderate the effectiveness of gratitude on conflict resolution, with its benefits being most pronounced in intimate, emotionally connected relationships.

As a result, the following hypothesis was established: H2: “Personal gratitude will influence positive conflict resolution styles in couple.”

### 1.3 Conflict resolution styles in couple and romantic love

One line of research finds that couples who employ constructive conflict resolution strategies—such as collaboration, compromise, and positive problem solving—report greater intimacy, satisfaction, and overall romantic love. For instance, Gottman and Levenson (2000) found that couples with more adaptive conflict behaviors (e.g., mutual repair attempts and softer start-ups) not only experienced fewer destructive interactions but also reported stronger emotional bonds and higher levels of romantic love. Similarly, Abreu-Afonso et al. (2021) conducting a study assessing conflict communication patterns in couples, found that couples who engaged in positive, cooperative conflict resolution—characterized by open communication, mutual repair attempts, and collaborative problem solving—reported higher levels of satisfaction and deeper emotional connection over time. The authors concluded that when partners adopt constructive conflict resolution styles, it not only reduces negative affect during disagreements but also reinforces a secure, supportive bond that deepens romantic connection.

In contrast, some studies have challenged a straightforward association between conflict resolution styles and romantic love. For example, research by Kurdek (1994) found that conflict resolution styles may have a direct impact on romantic love but moderated by factors such as individual personality, cultural expectations, and relationship context. In some samples, no clear association was found between the use of particular conflict resolution styles (e.g., avoidance or compromise) and overall romantic love ratings, suggesting. Additionally, Simpson et al. (1992) reported that while secure attachment is linked to effective conflict resolution, the mere adoption of a particular conflict style does not automatically translate into higher levels of romantic love, indicating that other relational processes (such as forgiveness, empathy, and shared meaning) may also be critical.

As a result, we established the following hypothesis: H3: “Conflict resolution styles in couple will positively influence romantic love.”

## 2 Research methodology

### 2.1 The present study

Although scientific studies are not unanimously clear regarding the associations between these main variables, it was presumed that religious partners express more frequently gratitude and solve more positively their conflicts which strengthen their romantic relationship. Thus, the aim of the present study was to investigate the relationship between gratitude and positive conflict resolution styles as mediators between religious faith, and romantic love.

Based on theoretical grounds and prior empirical evidence, the hypothesized relationships in this model are assumed to operate through a fully mediated pathway. Therefore, direct effects between non-adjacent constructs (e.g., faith to conflict resolution or faith to romantic love) were excluded to test the sequential and cumulative influence of each construct. Removing direct effects supports a more parsimonious model structure, minimizing unnecessary complexity while maintaining theoretical coherence.

The model uses circles to represent latent variables, reflecting underlying constructs assessed via multiple subscales, for which direct observational data are not available. The choice was to represent these constructs as latent variables rather than creating a single observed variable from summed subscale scores because latent variables allow us to account explicitly for measurement error and capture the shared variance among multiple subscales. This approach provides a more accurate and reliable representation of the underlying psychological construct compared to simply using summed scores as observed variables.

Thus, the study had three hypotheses:

H1: Religious faith will positively influence personal gratitude.
H2: Personal gratitude will influence positive conflict resolution styles in couple.
H3: Conflict resolution styles in couple will positively influence romantic love.

**Figure 1:**
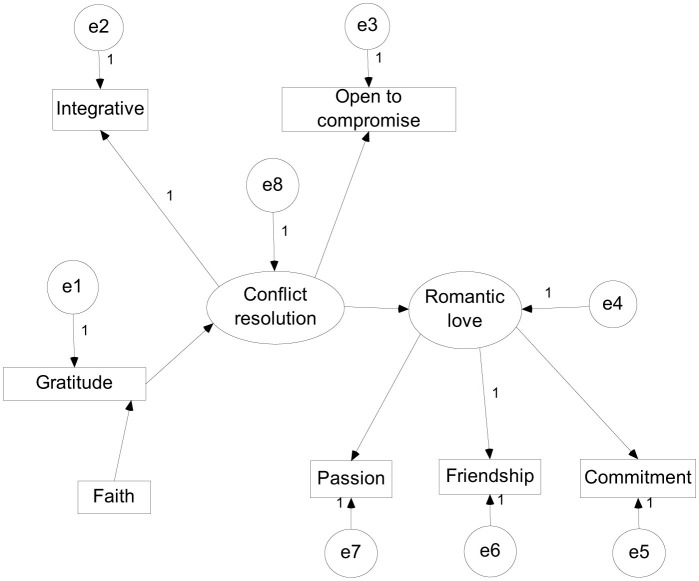
The presumed model of the relationships between the main variables of the research.

They were tested using Structural Equation Modeling in AMOS IBM SPSS (see Figure 1).

### 2.2 Participants

#### 2.2.1 Participants description

Participants consisted of married men and women who were not members of the same couple or family unit. The key characteristics of the participants are presented below (see Table 1). There were about 226 participants which according to some authors (Kline, 2023; Wolf et al., 2013) it is sufficient for SEM analysis.

**Table 1:** Key characteristics of the participants (*N* = 226).

**Variable**	**Descriptive statistics**	**Categories (frequency, %)**
Civil status	-	Married: (100%)
Age (years) *N = 219*	M = 40.67	(continuous variable)
	SD = 11.76	
	Range: 18–68	
Sex *N = 226*	–	Female: 123 (54.4%)
		Male: 103 (45.6%)
Background *N = 226*	–	Urban: 137 (60.6%)
		Rural: 89 (39.4%)
Years in Relationship *N = 224*	M = 17.51	(continuous variable)
	SD = 10.81	
	Range: 1–52	
Education *N = 226*	Coded 1–9	PhD: 3 (1.3%)
		College: 68 (30.1%)
		Faculty (non-graduate): 6 (2.7%)
		Master: 50 (22.1%)
		Post high school: 19 (8.4%)
		High school: 44 (19.5%)
		Professional school: 23 (10.2%)
		More than 8 classes but did not graduate high school: 9 (4.0%)
		8 or less classes: 4 (1.8%)
Children *N = 226*	Coded as 0 = no	0 children: 62 (27%)
	1 = yes	1 or > children: 164 (72%)
How many children *N = 225*	M = 1.48	0: 60 (26.5%)
	SD = 1.44	1: 56 (24.9%)
	Range: 0–12	2: 79 (35.1%)
		3: 20 (8.9%)
		4: 3 (1.3%)
		5: 3 (1.3%)
		6: 2 (0.9%)
		8: 1 (0.4%)
		12: 1 (0.4%)
Age of the small child (years) *N = 184*	M = 12.22	(continuous variable)
	SD = 9.81	
	Range: 0–40	
Age of the older child (years) *N = 167*	M = 14.46	(continuous variable)
	SD = 10.99	
	Range: 0–40	
Health *N = 225*	Coded 1–4	I am healthy: 191 (84.5%)
		Chronic illness: 30 (13.3%)
		Physical handicap: 1 (0.4%)
		Psychological illness: 3 (1.3%)
Income *N = 226*	Coded 1–5	Insufficient: 11 (4.9%)
		Less than needed: 19 (8.4%)
		As much as needed: 152 (67.3%)
		More than needed: 28 (12.4%)
		Much more than needed: 16 (7.1%)
Job *N = 226*	Coded 1–5	Working abroad: 15 (6.6%)
		Working in the country: 164 (72.6%)
		Not working: 24 (10.6%)
		Retired: 10 (4.4%)
		Student: 13 (5.8%)

For categorical variables, education was assessed via nine categories ranging from minimal schooling (coded as 1) to high school (coded as 9). Health was coded such that 1 = I am healthy, 2 = Chronic illness, 3 = Physical handicap, and 4 = Psychological illness. Income and job were measured using ordinal scales (1–5) with higher scores indicating greater income sufficiency and differing employment statuses, respectively. Some variables are continuous (with means, standard deviations, and ranges) and others are categorical (with frequencies and percentages).

#### 2.2.2 Participants access

The measures were put into Google Forms and the link was distributed based on the snowball method online. While the method ensured cultural alignment and trust, it likely introduced homogeneity (e.g., sampling devout, similar couples), and deepened insights into the reality studied. The post announced that the current research is seeking married respondents. As the measure was dedicated exclusively to married people there were no recorded declined invitations.

#### 2.2.3 Participants inclusion and exclusion

The entry page of the Google Forms had several screening questions referring to the existence of serious mental or physical health conditions that could affect the study. In these cases, the respondents were denied filling up the form.

### 2.3 Measurements

The *Santa Clara Strength of Religious Faith Questionnaire (SCSRFQ)* was developed by Plante and Boccaccini (1997) and published in *Pastoral Psychology*. This instrument is designed to measure the strength of an individual's religious faith in a way that is not confined to any one religious tradition. The SCSRFQ uses a 4-point Likert scale, ranging from “strongly disagree” to “strongly agree,” and comprises 10 items that form a unidimensional scale. A representative item is, “My religious faith is extremely important to me.” The questionnaire is intended for use across a variety of religious groups and has been applied in multiple contexts to explore the influence of faith on mental health and wellbeing. Recent investigations have included studies such as those by Sherman et al. (1999), which examined the link between religious faith and mental health in clinical samples, and Lewis et al. (2001), who conducted a confirmatory factor analysis to further validate the instrument's structure. In the present study was applied the Romanian version of the instrument. Alpha Cronbach for the scale was 0.957.

*Sternberg's Triangular Theory of Love Scale (STLS)* is grounded in Robert J. Sternberg's influential Triangular Theory of Love, which conceptualizes love as comprising three interrelated components: intimacy, passion, and commitment (Cassepp-Borges and Pasquali, 2012). First introduced in Sternberg's seminal 1986 article in *Psychological Review*, the STLS typically employs a 9-point Likert scale, where respondents rate items from “not at all” to “extremely.” The study used 12 items for each component. An example item addressing passion might be, “I feel a strong attraction for my partner.” The STLS is primarily used among individuals in romantic relationships to capture the multifaceted nature of love. Its widespread adoption in relationship research is evidenced by studies such as those by Sumter et al. (2013), which explored love dynamics in adolescent relationships, and by Acker and Davis (1992), who examined how different love styles impact overall relationship quality. The instrument was translated from English to Romanian following the scientific rigorous procedures. Alpha Cronbach for Friendship was 0.936, for passion was 0.954, and for commitment was 0.918.

The *Gratitude Questionnaire – 6 (GQ-6)* was developed by McCullough et al. (2002) first published in the *Journal of Personality and Social Psychology*. This instrument is designed to measure dispositional gratitude by assessing four dimensions: frequency (how often gratitude is experienced), intensity (the strength of the feeling), span (the range of gratitude triggers), and density (the number of people or entities toward whom one feels grateful). The study used 4 items from the GQ-6, each rated on a 7-point Likert scale ranging from “strongly disagree” to “strongly agree,” with two items reverse scored to control for response bias. An example item is, “I have so much in life to be thankful for.” Originally aimed at adults and adolescents, the scale has been successfully employed in diverse cultural settings. Recent studies, such as those by Disabato et al. (2017) and Boggiss et al. (2020), have confirmed the instrument's robust psychometric properties and have explored its relationship with mindfulness and overall psychological wellbeing. The instrument was translated from English to Romanian following the scientific rigorous procedures. Cronbach Alpha for the gratitude scale was 0.829.

The *ROCI-II, Conflict Resolution Behavior in Romantic Relationships* (Rahim, 1983) measures five distinct styles—Integrative, Dominating, Submissive, Avoiding, and Compromising—across 35 items (7 per subscale) rated on a 7-point Likert scale. An example item in English is: “I try to analyze a problem with my partner in order to find a solution acceptable to both of us.” The present study selected two subscales. The Integrative approach reflects a high concern for both one's own needs and the needs of others, while the Compromising approach involves a moderate concern for self and others. The two subscales were chosen for the present study as they keep an optimal balance between asking and receiving. The instrument was translated from English to Romanian following the scientific rigorous procedures. Cronbach Alpha for the integrative approach was 0.886, while for compromising approach 0.759.

### 2.4 Data collection methods

All questionnaires were written in Google Forms and thus data was collected online. Participants were selected based on accessibility. No reward was given to respondents. All data was collected voluntarily, and all participants could retreat from the study at any time, but this did not happen.

### 2.5 Data analysis techniques

The data were analyzed using structural equation modeling (SEM) in IBM SPSS AMOS software to test the hypothesized relationships between religious faith, gratitude, conflict resolution styles, and romantic love. Descriptive statistics and Pearson correlations were first computed in SPSS to examine baseline associations between variables, while AMOS facilitated the final SEM analysis, employing maximum likelihood estimation to ensure robust parameter estimates. Although AMOS refers to causal relationship diagram or to casual model of relationship, they are called “causal” as the software tests the directionality of the influence nor only the association.

Before the main analyses the data was imported into SPSS IBM 26. We analyzed the raw data, looked for atypical answers and find solutions like correcting or eliminating answers, we recoded data, sum up individual variables to form total scores and renamed variables.

### 2.6 Ethical considerations

The study adhered to stringent ethical guidelines to ensure participant rights and welfare. Prior to data collection, approval was obtained from an institutional review board, and all participants provided informed consent, clearly outlining the study's purpose, voluntary nature, and confidentiality protocols. Anonymity was preserved by de-identifying responses, and data were securely stored using encrypted digital platforms accessible only to the research team. Given the cultural sensitivity of discussing religious and relational dynamics in Romania's faith-oriented context, measures were taken to avoid coercive language and ensure participants felt no obligation to disclose personal beliefs or intimate details. Participants were informed of their right to withdraw at any stage without repercussions. These steps aligned with international ethical standards, fostering trust and minimizing risks in this culturally embedded research.

## 3 Results

In order to analyze the results, it was firstly performed a multiple Pearson Correlation, then the SEM analysis, we present the model comparison.

### 3.1 Correlation matrix

In order to investigate how the main variables of the research are related we performed a multiple Pearson correlation (see Table 2).

**Table 2:** Pearson correlations matrix between the main variables of the research.

**Variables**	**1**	**2**	**3**	**4**	**5**	**6**
1.Gratitude						
2. Faith	0.420^**^					
3. Commitment	0.109	0.106				
4. Passion	0.100	0.084	0.812^**^			
5. Friendship	0.178^**^	0.115	0.754^**^	0.787^**^		
6. Integrative	0.389^**^	0.179^**^	0.434^**^	0.448^**^	0.529^**^	
7. Open to compromise	0.292^**^	0.057	0.174^**^	0.190^**^	0.248^**^	0.557^**^

** significant at 0.01 (2-tailed).

In the correlation matrix, there are 21 unique pairwise comparisons among the seven variables. Out of these, 12 correlations are statistically significant at the 0.01 level. The highest significant positive correlation is between Commitment and Passion (r = 0.812), indicating a strong association between the subscales of the same measure. The lowest significant positive correlation is between Faith and Integrative style (r = 0.179), suggesting a weaker relationship.

### 3.2 SEM analysis

**Figure 2:**
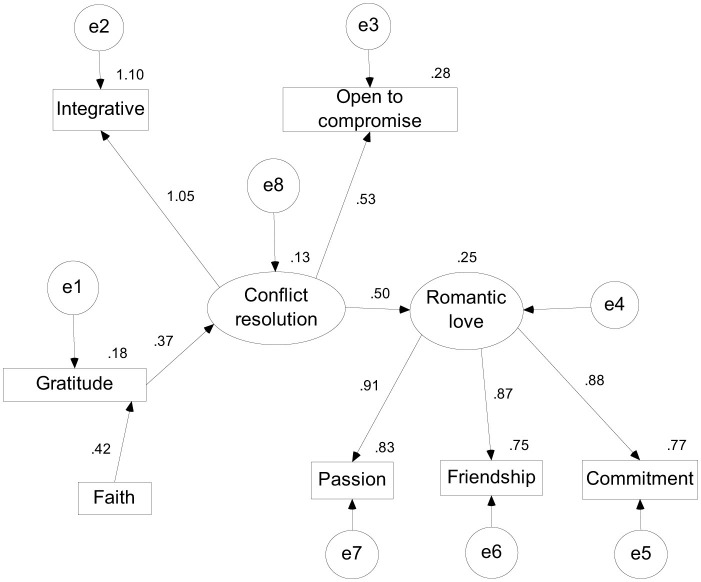
The trimmed model presenting the standardized regression weights and the multiple correlations.

The SEM analysis in AMOS indicated the following final model (see Figure 2).

The evaluation of your structural equation model's fit indices indicates a strong alignment with the observed data (see Table 3). The Chi-square (CMIN) value is 18.927 with 13 degrees of freedom, resulting in a CMIN/DF ratio of 1.456. This ratio is below the commonly accepted threshold of 2, suggesting an acceptable fit. The Goodness of Fit Index (GFI) is 0.976, and the Adjusted Goodness of Fit Index (AGFI) is 0.947; both values exceed the recommended cutoff of 0.95, indicating an excellent fit. The Root Mean Square Error of Approximation (RMSEA) is 0.045, which is below the 0.05 threshold, signifying a close fit. Additionally, the Comparative Fit Index (CFI) stands at 0.992, surpassing the 0.95 benchmark for an excellent fit. Collectively, these indices demonstrate that your model fits the data exceptionally well.

**Table 3:** Model fit indices.

**Fit index**	**Value**	**Interpretation**
Chi-square (CMIN)	18.927	A non-significant chi-square (*p* = 0.125) suggests a good fit.
Degrees of Freedom (DF)	13	-
CMIN/DF	1.456	Values <2 indicate a good fit.
Goodness of Fit Index (GFI)	0.976	Values ≥0.95 indicate an excellent fit.
Adjusted Goodness of Fit Index (AGFI)	0.947	Values ≥0.90 are acceptable.
Root Mean Square Error of Approximation (RMSEA)	0.045	Values ≤ 0.05 indicate a close fit.
Comparative Fit Index (CFI)	0.992	Values ≥0.95 indicate an excellent fit.

The interpretations are based on commonly accepted thresholds in structural equation modeling.

The regression weights indicated the following results (see Table 4).

**Table 4:** Regression Weights regarding the relationships between the main variables of the research.

**Dependent variable**	**Predictor**	**Standardized estimate (β)**	**Unstandardized estimate**	**SE**	**CR**	***p*-value**
Gratitude	Faith	0.420	0.218	0.031	6.951	<0.001
Conflict resolution	Gratitude	0.367	0.589	0.094	6.261	<0.001
Romantic love	Conflict resolution	0.504	0.887	0.163	5.451	<0.001
Friendship	Romantic love	0.867	1.000 (fixed)	-	-	-
Passion	Romantic love	0.912	1.401	0.077	18.299	<0.001
Commitment	Romantic love	0.880	1.208	0.069	17.451	<0.001
Integrative	Conflict resolution	1.047	1.000 (fixed)	-	-	-
Open to compromise	Conflict resolution	0.530	0.566	0.097	5.859	<0.001

In your model, all regression paths are statistically significant (*p* < 0.001), suggesting robust relationships between the variables. Notably, the path from Love to Passion has the highest standardized estimate (β = 0.912), indicating a strong association. Amos module provides modification indices, but as a matter of fact, the association between faith or gratitude and romantic love, and gratitude and romantic love were all not significant, thus this confirming, from a statistical point of view, the associations in the model presumed.

### 3.3 Model comparisons

The comparison from the presumed model and the trimmed model show significant differences (see Table 5).

**Table 5:** Comparisons of the presumed and trimmed models.

**Models**	**χ^2^**	**df**	**χ^2^/df**	***p***	**#variables**
Presumed	17.338	10	1.733	0.067	17
Trimmed	18.927	13	1.455	0.125	17

The analysis comparing the presumed and trimmed models revealed nuanced differences in model fit. The presumed model (χ^2^ = 17.338, df = 10, χ^2^/df = 1.733, *p* = 0.067) initially showed borderline statistical significance (*p* < 0.10) and a χ^2^/df ratio slightly above the ideal threshold of 1.5, suggesting moderate fit. After trimming non-significant paths and refining parameters, the trimmed model (χ^2^ = 18.927, df = 13, χ^2^/df = 1.455, *p* = 0.125) demonstrated improved fit indices: a non-significant chi-square (*p* > 0.05) and a χ^2^/df ratio below 1.5, aligning with excellent model fit standards. Notably, both models retained the same number of variables (17), indicating that trimming did not reduce complexity but instead optimized the structural relationships. The trimmed model's superior parsimony (lower χ^2^/df ratio and higher *p*-value) highlights the benefits of refining pathways to better reflect the data while maintaining theoretical coherence.

## 4 Discussions

The aim of the present research was to investigate the relationship between religious faith, gratefulness, positive conflict resolution styles and romantic love. The framework depicts a step-by-step progression where spiritual conviction fosters a sense of thankfulness, which then improves the ability to resolve disagreements, thereby deepening romantic bonds. This interconnected sequence highlights how personal values and emotional skills work together to influence partnership dynamics, emphasizing their combined impact rather than individual contributions. The results confirmed all three hypotheses presuming significant relationships between variables.

Referring to particular relationships, firstly, the strong positive association between religious faith and personal gratitude aligns with prior research emphasizing the role of intrinsic spirituality in fostering thankfulness. As posited by Emmons and Kneezel (2005), individuals who internalize religious values often perceive life's blessings as divine gifts, cultivating a sustained sense of gratitude.

The observation that intrinsic religiosity enhances gratitude more than extrinsic religiosity could stem from variations in religious capital—such as deeply held beliefs, personal emotional commitment, and active participation in religious practices. Another possible explanation ties into the concept of gendered emotional labor (Russell Hochschild, 2012), given that women frequently assume greater relational responsibilities in both religious and domestic contexts. Additionally, within Eastern Europe's evolving social and religious dynamics (Inglehart and Norris, 2003), this connection might function as a way to restore a sense of purpose and unity amid change. This connection was particularly pronounced in Romania's highly religious context, where spiritual practices like prayer and communal worship may amplify gratitude through shared rituals and reflection.

Second, gratitude's significant influence on cooperative conflict resolution corroborates evidence that thankful individuals prioritize relational harmony over adversarial tactics. As Dizon (2020) indicated, gratitude interventions foster empathy and openness, which are critical for collaborative problem-solving. The Romanian couples' reliance on adaptive strategies, such as integrative negotiation and compromise (Marici et al., 2023), may reflect how gratitude redirects focus from self-interest to mutual benefit. In intimate relationships, where emotional bonds are stronger, gratitude may more effectively buffer against destructive conflict patterns. This study extends prior findings by situating gratitude within a relational process linking it not just to conflict management but to broader emotional outcomes.

Finally, the robust link between adaptive conflict resolution and romantic love reinforces (Gottman and Levenson, 2000)'s assertion that constructive conflict behaviors—like open communication and mutual repair—strengthen emotional bonds (Marici, 2025). The Romanian sample's high correlation between commitment and passion (r = 0.812) suggests that collaborative conflict resolution may sustain both pragmatic and affective dimensions of love.

One limit of the study is the reduced sample size of the respondents. Although we did not limit the age intervals as investigated variables produce effects for all age groups, future research could consider narrower age categories and check for differences and investigate various cultural contexts too (Nadolu et al., 2020). While the current research employed a variable-centered approach to assess causal links between key factors, subsequent studies might benefit from person-centered methods, such as latent profile analysis, to uncover unique relational typologies or subgroup variations. This alternative lens could provide richer understanding of how faith, gratitude, and conflict resolution strategies intersect within different individuals or partnerships, revealing nuanced patterns beyond broad directional trends.

The model reflects a stepwise process where religious faith influences romantic love through gratitude and conflict resolution. Interpretation should emphasize this cumulative flow rather than isolated paths, as each variable builds upon the previous one.

## 5 Conclusions

The present study found that religious faith influences personal gratitude, which is positively associated with positive conflict resolution styles, which in turn leads to a higher score of romantic love.

As our study investigated intrinsic faith one question is whether relationships in the presumed model would maintain if faith was extrinsic motivated, which we speculate that would not. The cultural homogeneity of the Romanian sample—where religious engagement is often deeply personal—likely enhanced the observed effect, underscoring the importance of context in interpreting spirituality's role. Future cross-cultural studies could further disentangle how other types of societies, more pluralistic environments would moderate this dynamic.

In addition, future work should explore bidirectional effects, such as whether deepened romantic love further reinforces gratitude or religious engagement, creating a virtuous cycle of relational growth. Future research could explore longitudinal designs to test whether these effects persist over time or vary during relational stressors, as the present study focused on positive variables. For practitioners, the findings advocate training couples in gratitude and conflict skills as dual pathways to nurturing enduring love, particularly in religious communities where spirituality and relational harmony are intertwined.

## Data Availability

The datasets presented in this study can be found in online repositories. The names of the repository/repositories and accession number(s) can be found below: https://www.researchgate.net/publication/389581985_Database_for_study_Religious_Faith_Gratitude_Conflict_Resolution_Styles_and_Romantic_Love.

## References

[B1] Abreu-Afonso J. Ramos M. M. Queiroz-Garcia I. Leal I. (2021). How couple's relationship lasts over Time? A model for marital satisfaction. Psychol. Rep. 125, 1601–1627. 10.1177/00332941211000651 33736540 PMC9136471

[B2] Acker M. Davis M. H. (1992). Intimacy, passion and commitment in adult romantic relationships: a test of the triangular theory of love. J. Soc. Pers. Relation. 9, 21–50.10611781

[B3] Algoe S. B. Gable S. L. Maisel N. C. (2010). It's the little things: Everyday gratitude as a booster shot for romantic relationships. Pers. Relatsh. 17, 217–233. 10.1111/j.1475-6811.2010.01273.x

[B4] Bahnaru A. Runcan R. Runcan P. (2019). Religiosity and marital satisfaction. Rev. Asistentă Soc. 3, 107−114. Available online at: https://www.researchgate.net/publication/344624802_Religiosity_and_Marital_Satisfaction

[B5] Boggiss A. L. Consedine N. S. Brenton-Peters J. M. Hofman P. L. Serlachius A. S. (2020). A systematic review of gratitude interventions: effects on physical health and health behaviors. J. Psychosom. Res. 135:110165. 10.1016/j.jpsychores.2020.110165 32590219

[B6] Bowlby J. (1969). Attachment and Loss—Vol. 1: Attachment. New York: Basic Books.

[B7] Cassepp-Borges V. Pasquali L. (2012). Sternberg's Triangular Love Scale national study of psychometric attributes. Paidéia 22, 21–31. 10.1590/S0103-863X2012000100004

[B8] Disabato D. J. Kashdan T. B. Short J. L. Jarden A. (2017). What predicts positive life events that influence the course of depression? A longitudinal examination of gratitude and meaning in life. Cogn. Therap. Res. 41, 444–458. 10.1007/s10608-016-9785-x

[B9] Dizon M. T. S. (2020). The effect of gratitude journaling on conflict resolution in intimate dyadic relationships. Philippine J. Psychol. 53, 117–144. 10.31710/pjp/0053.05

[B10] Emmons R. A. Kneezel T. T. (2005). Giving thanks: spiritual and religious correlates of gratitude. J. Psychol. Christ. 24.

[B11] Fehr R. Fulmer A. Awtrey E. Miller J. A. (2017). The grateful workplace: a multilevel model of gratitude in organizations. Acad. Manage. Rev. 42, 361–381. 10.5465/amr.2014.0374

[B12] Gottman J. M. Levenson R. W. (2000). The timing of divorce: predicting when a couple will divorce over a 14-year period. J. Marr. Family 62, 737–745. 10.1111/j.1741-3737.2000.00737.x11924092

[B13] Huynh V. S. Tran-Thien G. P. Nguyen T. B. Nguyen X. T. K. Nguyen V. H. A. Tran-Chi V. L. (2024). What do we know about the influence of believers' religiosity on happiness and gratitude? A perspective for clinical practice. Psychol. Res. Behav. Manag. 2433–2447. 10.2147/PRBM.S465729 38912159 PMC11193991

[B14] Inglehart R. Norris P. (2003). Rising Tide: Gender Equality and Cultural Change Around the World. Cambridge: Cambridge University Press. 10.1017/CBO9780511550362

[B15] Kale P. Singh H. Perlmutter H. (2000). Learning and protection of proprietary assets in strategic alliances: building relational capital. Strat. Manag. J. 21, 217–237. 10.1002/(SICI)1097-0266(200003)21:3<217::AID-SMJ95>3.0.CO;2-Y

[B16] Kline R. B. (2023). Principles and Practice of Structural Equation Modeling. London: Guilford publications.

[B17] Komase Y. Watanabe K. Hori D. Nozawa K. Hidaka Y. Iida M.. (2021). Effects of gratitude intervention on mental health and well-being among workers: a systematic review. J. Occup. Health 63:e12290. 10.1002/1348-9585.12290 34762326 PMC8582291

[B18] Kraus R. Desmond S. A. Palmer Z. D. (2015). Being thankful: examining the relationship between young adult religiosity and gratitude. J. Relig. Health 54, 1331–1344. 10.1007/s10943-014-9923-2 25073767

[B19] Krause N. (2009). Religious involvement, gratitude, and change in depressive symptoms over time. Int. J. Psychol. Relig. 19, 155–172. 10.1080/10508610902880204 20333271 PMC2843928

[B20] Kurdek L. A. (1994). Conflict resolution styles in gay, lesbian, heterosexual nonparent, and heterosexual parent couples. J. Marriage Fam. 50, 705–722. 10.2307/352880

[B21] Lewis C. A. Shevlin M. McGuckin C. Navrátil M. (2001). The Santa Clara strength of religious faith questionnaire: confirmatory factor analysis. Pastor. Psychol. 49, 379–384. 10.1023/A:101037072854624892461

[B22] Marici M. (2025). Towards a new theory of couple functioning. OSF. 10.31234/osf.io/ypkw2_v3 PMC12679299 41356053

[B23] Marici M. Clipa O. Schipor M. D. Runcan R. Andrei A. M. (2023). Offering and asking for help with domestic chores in couple relationships. Int. J. Environ. Res. Public Health 20:3708. 10.3390/ijerph20043708 36834402 PMC9961060

[B24] McCullough M. E. Emmons R. A. Tsang J. A. (2002). The grateful disposition: a conceptual and empirical topography. J. Pers. Soc. Psychol. 82:112. 10.1037//0022-3514.82.1.112 11811629

[B25] Mikulincer M. Shaver P. R. (2010). Attachment in Adulthood: Structure, Dynamics, and Change. London: Guilford Publications.

[B26] Nadolu D. Runcan R. Bahnaru A. (2020). Sociological dimensions of marital satisfaction in Romania. PLoS ONE 15:e0237923. 10.1371/journal.pone.0237923 32817661 PMC7446780

[B27] Nahapiet J. Ghoshal S. (1998). Social capital, intellectual capital, and the organizational advantage. Acad. Manag. Rev. 23, 242–266. 10.2307/259373

[B28] Plante T. G. Boccaccini M. (1997). Reliability and validity of the Santa Clara strength of religious faith questionnaire. Pastoral Psychol. 45, 429–437. 10.1007/BF0231064311536422

[B29] Rahim M. (1983). A measure of handling interpersonal conflict. Acad. Manag. J. 26, 368–376. 10.2307/255985 10263067

[B30] Russell Hochschild A. (2012). The Managed Heart: Commercialization of Human Feeling. New York: University of California Press. 10.1525/9780520951853

[B31] Sandage S. J. Hill P. C. Vaubel D. C. (2011). Generativity, relational spirituality, gratitude, and mental health: relationships and pathways. Int. J. Psychol. Relig. 21, 1–16. 10.1080/10508619.2011.532439

[B32] Sherman A. C. Plante T. G. Simonton S. Adams D. C. Burris S. K. Harbison C. (1999). Assessing religious faith in medical patients: cross-validation of the Santa Clara Strength of Religious Faith Questionnaire. Pastor. Psychol. 48, 129–141. 10.1023/A:1022094727122

[B33] Simpson J. A. Rholes W. S. Nelligan J. S. (1992). Support seeking and support giving within couples in an anxiety-provoking situation: the role of attachment styles. J. Pers. Soc. Psychol. 62, 434. 10.1037/0022-3514.62.3.434

[B34] Sumter S. R. Valkenburg P. M. Peter J. (2013). Perceptions of love across the lifespan: differences in passion, intimacy, and commitment. Int. J. Behav. Dev. 37, 417–427. 10.1177/0165025413492486

[B35] Watkins P. C. Frederick M. Davis D. E. Emmons R. A. (2024). Exploring the cognitive context of gratitude to God: emotional impact and appraisals of benefits from God. J. Posit. Psychol. 19, 166–182. 10.1080/17439760.2023.2230458

[B36] Wolf E. J. Harrington K. M. Clark S. L. Miller M. W. (2013). Sample size requirements for structural equation models: an evaluation of power, bias, and solution propriety. Educ. Psychol. Meas. 73, 913–934. 10.1177/0013164413495237 25705052 PMC4334479

